# Why Open Drug Discovery Needs Four Simple Rules for Licensing Data and Models

**DOI:** 10.1371/journal.pcbi.1002706

**Published:** 2012-09-27

**Authors:** Antony J. Williams, John Wilbanks, Sean Ekins

**Affiliations:** 1 Royal Society of Chemistry, Wake Forest, North Carolina, United States of America; 2 Consent to Research, Oakland, California, United States of America; 3 Collaborations in Chemistry, Fuquay-Varina, North Carolina, United States of America; University of California San Diego, United States of America

## Abstract

When we look at the rapid growth of scientific databases on the Internet in the past decade, we tend to take the accessibility and provenance of the data for granted. As we see a future of increased database integration, the licensing of the data may be a hurdle that hampers progress and usability. We have formulated four rules for licensing data for open drug discovery, which we propose as a starting point for consideration by databases and for their ultimate adoption. This work could also be extended to the computational models derived from such data. We suggest that scientists in the future will need to consider data licensing before they embark upon re-using such content in databases they construct themselves.

## Introduction

Public online databases [1] supporting life sciences research have become valuable resources for researchers depending on data for use in cheminformatics, bioinformatics, systems biology, translational medicine, and drug repositioning efforts, to name just a few of the potential end user groups. Worldwide funding agencies (governments and not-for-profits) have invested in public domain chemistry platforms. In the United States these include PubChem [2], ChemIDPlus [3], and the Environmental Protection Agency's ACToR [4], while the United Kingdom has funded ChEMBL [5] and ChemSpider [6], among others, and new databases continue to appear annually [7].

We have argued recently that the data quality contained within many of these databases is suspect [8] and scientists should consider issues of data quality [9] when using these resources. By assimilating various data sources together and meshing data on drugs, proteins, and diseases, these various databases and network and computational methods may be useful to accelerate drug discovery efforts. The development of related cheminformatics platforms or derived models without care given to data quality is a poor strategy for long-term science [10] as errors become perpetuated in additional databases. There is real evidence that the integration of large, heterogeneous sets of databases and other types of content is “unreasonably effective” at accelerating the conversion of data into knowledge [11]. This implies the need for technical and semantic work to bring databases together that were never designed for interoperability [12], which is in itself a significant task [13], [14].

As we and others have argued previously, there is another dimension to interoperability than technical formats [12] and ontological agreement [15]: the complex interactions of database licenses and terms of use around intellectual property. Many of these online databases have either obscure or confused licensing terms [16], and even in those cases where data are freely available for download and reuse there are often no clear definitions. Many databases simply “cut and paste” prohibitive copyright schema from traditional websites, or fail to address download and reintegration entirely (*ibid*). Since copyright law requires explicit permissions in advance to make use of copyrighted works, it is certainly unsafe to assume data licensing rights for any database that does not explicitly allow it.

The availability of data for download and reuse is an important offering to the community, as these data may be used for the purpose of modeling to develop prediction tools [17]. In addition, data can be ingested into internal systems inside pharmaceutical companies to mesh with their existing private data [18], including in the expanding Linked Open Data cloud or in freely available online databases, and can be downloaded and used to enhance their content and to establish linking between data. The Open PHACTS project [19], [20] utilizes a semantic web approach to integrate chemistry and biology data across a myriad of data sources, including for chemistry ChEBI, ChEMBL, and DrugBank, and for biology UniProt, Wikipathways, and many others. The chemical structure representations are obtained from ChemSpider, which has previously imported the chemical databases and standardized according to their data model and are making the data available as open data to the project. Many of the primary online databases already have multiple links to external systems. This linking may be achieved by using available database services to form transitory links in by, for example, using a chemical representation such as an InChI [21] to probe an application programming interface, search for the compound, and generate the linking URL in real time. Commonly, however, the links are more permanent in nature and are generated by downloading data from the various data sources, depositing a subset of the data (generally the chemical compound and associated database identifier), and using the particular database URL structure to form permanent links. This act of download and deposition of multiple data sources is commonly mixing the various licenses, if licenses are even declared, which, in many cases, they are not.

In some ways, there are analogous difficulties in the exchange of computational models like quantitative structure activity relationship (QSAR) datasets [22]—while there are efforts to standardize how the data and models are stored, queried, and exchanged, there has been little consideration of licenses required to enable making the sharing of open source models a reality [23]. Similarly, one could consider the creation of maps of disease and how they are shared and reused [24] in the same manner.

The potential legal fragility of knowledge products derived from online databases with poorly understood licensing for each of the databases is a real problem, and one that will only increase in severity over time. This realization is not novel; indeed, the chemical blogosphere has been host to many discussions regarding the need for clear data licensing definitions on chemistry-related data. Many scientists likely echo these comments, but we will provide some examples. In particular, Peter Murray-Rust [25] espouses the value of “open data” [26] to the scientific discovery process and encourages clear licensing of all chemistry data according to Open Knowledge Definition (OKD) [27] and the Panton Principles [28].

Herein we provide an extensive background to the intellectual property around data and databases in the sciences involved in drug discovery, those of biology, chemistry, and related fields, as well as discussion of open data licensing, openness, and open license limitations (Text S1). More importantly, we provide a set of rules that practitioners might apply when making data or databases available via the Internet or mobile apps [29]. Our ultimate goal is to illuminate the legal fragility of the database ecosystem in the drug discovery sciences, and to initiate a conversation about creating best practices.

## Simple Rules for Licensing “Open” Data

We suggest based on our analysis of the current data situation (Text S1) the ideal is to use strong default rules for openness. From a copyright and database rights perspective, the public domain gives the most clarity and should be the default setting for data deposit, although it may not always be achievable. Understanding this is vital, because it sets the bar at the right height. Justifications for additional controls should be subject to argument—one often finds those controls are unnecessary when the discussion is framed this way.

It is also important to avoid noncommercial or share-alike approaches whenever possible. These are attractive terms to many data providers, but create significant barriers to interoperability. Noncommercial data might be incompatible for researchers at a pharmaceutical company, even to run a simple web-based query. It is important to realize data under a share-alike license from one entity is probably not combinable with data under a share-alike license from another entity (this lack of interoperability kept Creative Commons licensed images out of Wikipedia for years, and is not one we wish to introduce into the ecosystem again!).

Thus, we propose the following simple rules for developing data licensing approaches inside scientific projects.

1. Before you begin a database project, convene a meeting of all of the stakeholders. Expose all of the expectations of the group and decide if your goals are primarily scientific, commercial, or mixed. If mixed, take a stern look at the actual commercial potential of the project. Invite technology transfer offices to join you—they have greater experience in the realities of commercialization.
2. If your project is scientific in nature, and not commercial, explore the benefits of open licensing and drawbacks of enclosure. Go through the various definitions and find the most common ground possible, always placing the burden of proof on those who want more control and not less. This will create less “default enclosure” but allow for those increasingly rare situations in which “open” is not appropriate. Attempt to hew as closely as possible to the admittedly rigorous open definitions and standards, and do not write your own intellectual property licenses—instead, use existing and well deployed ones.
3. Develop simple explanations of your terms of use, and make them easy to find for users. Make sure that your licensing, expectations for attribution, terms of use, and more are linked in many ways to your data and database. Do not expect your users to read the legal text of your terms and conditions and licenses; instead, create simple summaries with linkages to the detailed text for users to access. Whenever possible, use metadata to indicate the licensing terms explicitly—the Creative Commons Rights Expression Language [30] is a good tool for this.
4. Don't ever lock up metadata. A significant swath of data will be incompatible with an open regime, whether it's to protect trade secrets or patient privacy. But the metadata that describes closed data, and how to access closed data, can be almost as valuable. If you can't make the data public domain, make the metadata public domain.

As a general rule, these four simple rules should allow us to build a more stable data and model sharing ecosystem while we live with some uncertainties until the courts rule on where the line of property stops and starts. We can't wait for the certainty to emerge, but we also want our systems to work when the courts do finally rule on issues such as where data and metadata stop and start, where copyright attaches, how data rights really affect re-use, and what it means to move towards a “cloud world” where copies aren't made of data at all. Following these heuristics when providing and/or accepting data is an approach that creates at least the opportunity to be forward-compatible for the future development of technologies.

But it is also important to pay close attention to licensing *sanitation* as a data consumer and user. No matter how tempting it is, do not copy a batch of informally open, but formally closed, data, run a database integration, and release the new database as “open”—that hurts the community. Instead, look for the terms of use, ask if it is “open”, post your enquiry, and only when you are certain, redistribute. We think databases funded by the government should at the very least be open, and if not this should be stated prominently.

## Conclusions

Although most scientists are likely unaware of this at present, data licenses are going to become increasingly important in science in the future, especially as we see more scientists embracing open notebook science, open science, and open-access publishing, and funding bodies promoting the increased accessibility of the fruits of their funding. We are likely not too far from funding bodies mandating immediate release of all data and results produced by each of their grantees, which is something we would advocate as potentially disruptive in its own right (S. Ekins et al., unpublished data).

We can hence imagine a near future in which many scientists will blog some or all of their research results while data aggregators will in turn consume this content and repackage it for others [31]. The licensing of this and other data will need to be clear if we are to build on the shoulders of giants and not have to face legal battles that pit Davids versus Goliaths. Considering data licensing as a part of the “scientific process” is vital for its future usability, and we strongly encourage scientists to consider data licensing before they embark upon re-using such content in databases they construct themselves or in the course of their research.

The four simple rules we have formulated for licensing data for open drug discovery represent a proposed starting point for consideration by database producers. These licenses could equally be used by individual scientists on their blogs and other online environments or accounts in which they make their data and models available for others.

## Supporting Information

Text S1 This consists of a discussion in three sections:
• Intellectual property rights in data: Copyright and Database Rights.
• Trends in legal certainty: Open Data Licensing.
• “Informal” Openness and Open License Limitations.
(PDF)

## References

[1] Williams AJ, Tkachenko V, Lipinski C, Tropsha A, Ekins S (2009) Free online resources enabling crowdsourced drug discovery. Drug Discovery World 10, Winter 33–38.

[2] National Center for Biotechnology Information (n.d.) The PubChem database. Available: http://pubchem.ncbi.nlm.nih.gov/. Accessed August 2012.

[3] US National Library of Medicine (n.d.) ChemIDPlus Advanced. Available: http://chem.sis.nlm.nih.gov/chemidplus/. Accessed August 2012.

[4] Judson R, Richard A, Dix D, Houck K, Elloumi F, et al (2008) ACToR–Aggregated Computational Toxicology Resource. Toxicol Appl Pharmacol 233: 7–13. 18671997 10.1016/j.taap.2007.12.037

[5] EMBL-EBI (n.d.) ChEMBL. Available: http://www.ebi.ac.uk/chembldb/index.php. Accessed August 2012.

[6] Pence H, Williams AJ (2010) ChemSpider: an online chemical information resource. J Chem Educ 87: 1123–1124.

[7] Galperin MY, Cochrane GR (2011) The 2011 Nucleic Acids Research Database issue and the online Molecular Biology Database Collection. Nucleic Acids Res 39: D1–D6. 21177655 10.1093/nar/gkq1243 PMC3013748

[8] Williams AJ, Ekins S, Tkachenko V (2012) Towards a gold standard: regarding quality in public domain chemistry databases and approaches to improving the situation. Drug Discov Today 17: 685–701. 22426180 10.1016/j.drudis.2012.02.013

[9] Williams AJ, Ekins S (2011) A quality alert and call for improved curation of public chemistry databases. Drug Disc Today 16: 747–750. 10.1016/j.drudis.2011.07.007 21871970

[10] Fourches D, Muratov E, Tropsha A (2010) Trust, but verify: on the importance of chemical structure curation in cheminformatics and QSAR modeling research. J Chem Inf Model 50: 1189–1204. 20572635 10.1021/ci100176x PMC2989419

[11] Halevy A, Norvig P, Pereira F (2009) The unreasonable effectiveness of data. Intelligent Systems 24: 8–12.

[12] Sansone SA, Rocca-Serra P, Field D, Maguire E, Taylor C, et al (2012) Toward interoperable bioscience data. Nat Genet 44: 121–126. 22281772 10.1038/ng.1054 PMC3428019

[13] NeuroCommons (n.d.) NeuroCommons project. Available: http://neurocommons.org. Accessed August 2012.

[14] Ruttenberg A, Rees JA, Samwald M, Marshall MS (2009) Life sciences on the Semantic Web: the Neurocommons and beyond. Brief Bioinform 10: 193–204. 19282504 10.1093/bib/bbp004

[15] Hastings J, Chepelev L, Willighagen E, Adams N, Steinbeck C, et al (2011) The chemical information ontology: provenance and disambiguation for chemical data on the biological semantic web. PLoS ONE 6: e25513 doi:10.1371/journal.pone.0025513. 21991315 10.1371/journal.pone.0025513 PMC3184996

[16] de Rosnay MD (2008) Check your data freedom: a taxonomy to assess life science database openness. Nature Precedings Available: http://dx.doi.org/10.1038/npre.2008.2083.1. Accessed August 2012.

[17] Ekins S, Williams AJ (2010) Precompetitive preclinical ADME/Tox Data: set it free on the web to facilitate computational model building to assist drug development. Lab on a Chip 10: 13–22. 20024044 10.1039/b917760b

[18] Zhu Q, Lajiness MS, Ding Y, Wild DJ (2010) WENDI: a tool for finding non-obvious relationships between compounds and biological properties, genes, diseases and scholarly publications. J Cheminform 2: 6. 20727184 10.1186/1758-2946-2-6 PMC2933596

[19] Azzaoui K, Jacoby E, Senger S, Rodríguez EC, Loza M, et al (2012) Analysis of the scientific competency questions followed by the IMI OpenPHACTS consortium for the development of the semantic web-based molecular information system OPS. Drug Disc Today In press.

[20] Williams AJ, Harland L, Groth P, Pettifer S, Chichester C, et al (2012) Open PHACTS: semantic interoperability for drug discovery. Drug Discov Today In press. Available: http://dx.doi.org/10.1016/j.drudis.2012.05.016. Accessed August 2012. 10.1016/j.drudis.2012.05.016 22683805

[21] Wikipedia (n.d.) InChIKey on the InChI Wikipedia page. Available: http://en.wikipedia.org/wiki/International_Chemical_Identifier#InChIKey. Accessed August 2012.

[22] Spjuth O, Willighagen EL, Guha R, Eklund M, Wikberg JE (2010) Towards interoperable and reproducible QSAR analyses: exchange of datasets. J Cheminform 2: 5. 20591161 10.1186/1758-2946-2-5 PMC2909924

[23] Gupta RR, Gifford EM, Liston T, Waller CL, Bunin B, et al (2010) Using open source computational tools for predicting human metabolic stability and additional ADME/TOX properties. Drug Metab Dispos 38: 2083–2090. 20693417 10.1124/dmd.110.034918

[24] Derry JM, Mangravite LM, Suver C, Furia MD, Henderson D, et al (2012) Developing predictive molecular maps of human disease through community-based modeling. Nat Genet 44: 127–130. 22281773 10.1038/ng.1089 PMC3643818

[25] Murray-Rust P (n.d.) Dr Peter Murray-Rust. Available: http://www.ch.cam.ac.uk/person/pm286. Accessed August 2012.

[26] Wikipedia (n.d.) Open data. Available: http://en.wikipedia.org/wiki/Open_data. Accessed August 2012.

[27] Open Knowledge Foundation (n.d.) Open data licensing. Available: http://wiki.okfn.org/Open_Data_Licensing. Accessed August 2012.

[28] Murray-Rust P, Neylon C, Pollock R, Wilbanks J, Open Knowledge Foundation Working Group on Open Data in Science (2010) The Panton principles. Available: http://pantonprinciples.org/. Accessed August 2012.

[29] Williams AJ, Ekins S, Clark AM, Jack JJ, Apodaca RL (2011) Mobile apps for chemistry in the world of drug discovery. Drug Disc Today 16: 928–939. 10.1016/j.drudis.2011.09.002 21924376

[30] Creative Commons (n.d.) ccREL: Creative Commons rights expression language. Available: http://www.w3.org/Submission/ccREL/. Accessed August 2012.

[31] Ekins S, Clark AM, Williams AJ (2012) Open drug discovery teams: a chemistry mobile app for collaboration. Molecular Informatics In press. doi:10.1002/minf.201200034. 10.1002/minf.201200034 PMC3503260 23198003

