# Supplementary material for: Why Open Drug Discovery Needs Four Simple Rules for Licensing Data and Models
Source: PLoS Comput Biol. 2012 Sep 27;8(9):e1002706. doi: 10.1371/journal.pcbi.1002706 (PMC3459841; doi:10.1371/journal.pcbi.1002706)
Supplement: Text S1 — This consists of a discussion in three sections: • Intellectual property rights in data: Copyright and Database Rights. • Trends in legal certainty: Open Data Licensing. • “Informal” Openness and Open License Limitations. (PDF) [file pcbi.1002706.s001.pdf]

Supplemental Information

Why Open Drug Discovery Needs Four Simple Rules for Licensing Data and Models

Antony J. Williams[1*], John Wilbanks[2] and Sean Ekins[3]

[1] Royal Society of Chemistry, 904 Tamaras Circle, Wake Forest, NC-27587, U.S.A.

[2] Consent to Research, Oakland CA USA

[3] Collaborations in Chemistry, 5616 Hilltop Needmore Road, Fuquay-Varina, NC 27526, U.S.A.

* To whom correspondence should be addressed. Antony Williams, E-mail: tony27587@gmail.com, Phone: +1 919 201 1516 John Wilbanks E-mail: jtw@del-fi.org Sean Ekins, E-mail address: ekinssean@yahoo.com, Phone: +1 215 687 1320

Text S1

Intellectual property rights in data: Copyright and Database Rights

Intellectual property rights covering data differ wildly depending on where one sits in the world. Copyrights and database rights, though they govern an international technical space, are still deeply rooted in geography and national implementations of legislation and treaties. It is generally the case that individual data points do not receive the protection of copyright but that data collections may be copyrighted. But has anyone considered the situation for data-driven works derived from such collections, such as computational models? Generally speaking, copyright attaches to creative "works" but not to ideas or facts. Thus, for example, a paper describing the tensile strength of steel, as a creative work, receives protection under copyright, but the fact that the measure itself is 400 megapascals does not. Neither can a chemical connection table, InChI or SMILES string [1] as a representation of a molecule be protected.

However, a depiction of the chemical compound as a figure in a publication *can* be copyrighted, if the depiction is creative enough to qualify as a "work" in and of itself, though that copyright is unlikely to prevent the independent creation of a similar figure from the underlying data. The intersection of social media and this point have led to several confusing situations in science blogging. And a data-driven computational model might receive one copyright for its software code, another copyright for the paper that describes the model, and outputs such as *predicted data* exist in a limbo of potential control and coverage.

To make things more complex, new services are emerging at a rapid pace that are bringing data online in unexpected ways. One of particular note is a new web-based resource called FigShare [2]. FigShare facilitates making figures open and available to the community. In theory, if authors uploaded their figures (containing molecules) to FigShare (or other online storage such as the Flickr [3] photo sharing service) prior to publication, even following copyright transfer to publishers, then near identical images will be available to be sourced from the internet. And the impact will only increase, as the figures in current scholarly papers are only a tiny percentage of all figures generated in experimental research - as the rest of the figures can not only be published, but cited and added to tenure applications, we can expect an explosion in data figures on the web unlinked to the traditional publishing ecosystem. Similarly, with ways to create nanopublications [4], excerpting data and molecules from papers via social media (e.g. Twitter) that can in turn be aggregated by mobile apps such as Open Drug Discovery Teams (ODDT) [5], to circumvent the original source .

The FigShare [2] example is the simplest case. We know that the file with the figure carries at least some copyright, as it’s been “fixed” in a medium and at least some selection and arrangement was involved in rendering the data into a figure. It is far too difficult to define where data transitions to become a copyrightable collection as a general rule. For example, we might ask if a file containing 100 chemical structures, associated chemical identifiers and experimental parameters such as melting points is

copyrightable. The answer is that the file itself probably will be, but the data contained within the file may still be extractable without violating copyright.

Based on the activities of commercial businesses in this domain, it is likely that some would assert the answer is yes (entities with commercial or other interests in the data) and that others would assert no (researchers and “open science” advocacy groups). But the answer is actually unknown. Until the courts begin to rule on the moment at which copyright attaches, we will continue to live in a world of contrary assertions of copyright and data. To create a more certain control, it is quite common in “traditional” publishing to attach contractual language to copyright licenses precisely to prevent this kind of extraction and to apply technological protection measures such as digital rights management to enforce those preventions.

Another complicating factor is the existence in some countries - but nowhere near a majority - of new intellectual property rights created in the past 20 years specifically to protect databases. Known as “database rights,” they are an example of *sui generis* intellectual property rights, created specifically to address an area outside of traditional IP rights, just as similar rights exist for fashions in France, or ship hull designs in the United States. These database rights are best known from the European Union, but are beginning to leak out to other countries, both via bilateral trade agreements with the EU or, ironically, via “open source database” licenses that propagate the right outside the shores of the EU as part of an attempt to make derived data works open (as in the case of the software models’ predictive data outcomes).

Database rights suffer from the same problem as copyright, however. Laws written even as recently as the 1990s have been badly left behind by technological advances. We do not have legal certainty as to where database rights begin and end. The EU right, for example, is triggered on “substantial” extraction of data from a database. But we do not know what “substantial” means in any given case, not to mention what a right based on the prevention of copying of data inside databases means in a world where queries run in a federated fashion on databases stored in the cloud and may be accessed by scientists using mobile apps residing in any country in the world.

It is entirely plausible that scientists will be able to legally circumvent preventions based on control of copying (whether database rights or copyrights) with clever technical design, and until the courts rule, we will not know the boundaries of the law. This uncertainty is the core source of the legal fragility mentioned in the introduction: we simply do not know the extent to which copyright protects data, which also means we do not know the extent to which we can use copyright either to enclose data or protect its public domain nature.

Trends in legal certainty: Open Data Licensing

The solution most commonly suggested to address the fragility is to embrace “openness” as a licensing strategy for scientific databases. The rationale is that open licenses on databases create freedom to operate for users to make use of both the data

inside a database and the entire database itself, which lowers the transaction costs on computational experimentation and recombination.

This is an idea that has gained traction in the self-identified open science community, unsurprisingly, but also in other quarters: GSK, for example, not only elected to publicly deposit its Tres Cantos Antimalarial (TCAMS) data set [6] in the ChEMBL-NTD, PubChem and Collaborative Drug Discovery Databases, it used the Creative Commons Zero (CC0) legal tool to do so - placing the data unambiguously into the public domain from a copyrights perspective [7]. Eli Lilly has followed a similar path with its TB Commons [8] initiative, using CC0 as part of an open innovation strategy. Pharmaceutical companies have also made software open source (e.g. J&J TransMart [9,10]) and co-sponsored efforts like the Pistoia Alliance [11,12] as pre-competitive efforts to share software and development costs.

The open data movement in science follows on the heels of open movements in software (Free/Libre Open Source Software, or FLOSS) and culture (Free Culture and Creative Commons). These movements have created vast public goods by turning copyright inside out. Open movements use licenses to guarantee openness, imposing only certain behaviors in users: you can copy my works, but only if you give me credit (“Attribution”) or only if you share your copies and changes under the same terms (“Copyleft” or “Share Alike”) [13].

The open data movement is a bit different than software and culture, though clearly influenced by them. First, there is no legal uncertainty over the role of copyright in software and creative works - it clearly attaches, protects, and can be licensed in ways that the courts recognize as legally binding. But data in many countries is in the public domain, and databases themselves are in many places only lightly touched by copyright, while database rights are themselves not widely implemented. Second, data is epistemologically different from a creative work or software - especially in the sciences, where the data are held to be “the facts” and thus something upon which to build rather than something to be enclosed.

These two differences color the emerging open data movement - particularly government data [14] in the United States and Europe. But science databases in general, and chemistry databases in particular, are subject to further pressures and complexities that raw government data about weather, for example, are not. Making them “open” from a legal perspective can be far more complex than making a research article open (which is more often a matter of business models and economics than law).

One set of complexities arises from the differing state of property laws over data mentioned above. But another can emerge from the variety of definitions of what “open” really means and the tools that database owners use to signal their intents. The Panton Principles [15] are perhaps the foremost attempt to bring clarity to the situation, stating that the banner of “open” should not be claimed in the sciences unless the data are explicitly placed in the public domain via a tool such as CC0.

But “open” is a word with different meanings and interpretations depending on context. The word itself comes pre-loaded with emotional tones for many software engineers, believing “free” is superior to “open,” whereas in the movement to create access to scholarly publications, “open” is superior to “free.” Part of the problem comes from the two meanings of free - in one sense as in “free of cost” and in another as in “freedoms” - which is why when discussing free software the preferred nomenclature is “free/libre” to make clear that freedom is the goal, not simply the absence of cost.

The Open Knowledge Foundation (OKF) in 2005 created and has promulgated the Open Knowledge Definition [14] (OKD) as a foundational set of principles for judging whether or not a piece of knowledge is open. The OKD defines knowledge as content, data, and government information, and covers rights of access, reuse, redistribution, absence of technical restriction, attribution, and more requirements to gain the “open” label. Putting aside epistemology (many scientists and philosophers would argue the idea of data being, itself, sufficient to be called knowledge, and most government information is itself either data or content!) the OKD serves as a handy Occam’s razor to judging openness versus enclosure [16]

To receive an OKD-compliant label, a dataset must not only be on the web free of charge, but must carry an explicit license granting the rights mentioned above. The attachment of an explicit license itself removes the uncertainty, even if the license is considered “closed” or is a liberal license that fails part of the definition (such as a

Creative Commons Non-Commercial or No-Derivatives license [17]). However, the reality is that many databases and datasets are simply placed on the web with no explicit license of any kind, and reasonable parties should therefore assume there are no rights to copy, reuse, and redistribute the data and databases in the absence of an explicit, OKD-compliant license to do so.

The OKD however is a general definition as noted above, covering a wide swath of works and objects. In the sciences, a more context-specific definition has been proposed (by a group that includes an author of this paper). Known as the "Panton Principles [15]," the definition calls for not just explicit licenses compliant with the OKD, but for the data to be explicitly placed in the public domain as the ideal. This is the status that is the most clear, most stable, least fragile and which can do public good [18].

It is also vital to remember that drug discovery data is subject to legal pressures beyond those of many other databases. The OKD and similar definitions (such as the Free Cultural Works definition) tend to orient themselves around copyright and database rights. But data and databases in drug discovery by definition relate to the discovery of drugs, and thus are often subject to trade secrecy, so that patents might be filed on the very non-digital compounds that emerge from the science.

We are seeing efforts due to fragmentation of the pharmaceutical industry to engage academic, industry as well as others like consortia in collaboration. This presents

additional challenges around privacy and IP across these diverse groups. The development of ‘cloud based’ collaborative platforms for drug discovery like CDD and HEOS offer flexible privacy and ways to share data that will fit differing types of collaborations.[19]

In the case of CDD and their ‘cloud-based’ ilk their role is to provide the software without wishing to retain any IP in the discoveries made by the scientists that use the software. In addition, in an effort to reach out to the scientific community CDD have made nearly 90 different datasets publically searchable and outside the private vaults. These include many libraries of small molecules screened against neglected diseases, an area where there is a greater willingness towards collaboration. Such ‘cloud-based’ collaboration tools in some ways may be pushing the envelope. First companies and organizations had to get comfortable with having their valuable IP residing on the cloud and outside their firewalls. This in itself for small companies is less of a problem as they do not have the internal informatics investments, but for pharmaceutical companies they have generally experimented with these tools through consortia type collaborations (tuberculosis and malaria). As pharmaceutical companies now have so many external collaborations the scale demands collaborative informatics tools to deal with CROs, academics etc. to manage the compounds, data and intellectual property. Ultimately we may see an explosion of the next generation of such tools.

Further complicating matters, data emerging from the study of drugs in patients is, as a rule, subject to requirements to de-identify or anonymize data, or to pass on

downstream obligations to protect confidentiality to users. These privacy requirements create obligations on data and databases that are often completely orthogonal to licenses designed for intellectual property. Indeed, one should probably assume that privacy rights, especially on data collected from individuals, can serve as a trump card to keep data closed even if all involved wish to license its relevant intellectual property rights openly.

“Informal” Openness and Open License Limitations

The trends in open licensing we describe above are far from the norms of practice in science at large, much less in drug discovery. Most in the community have no clear consensus on what “open means”, which is a reflection of the variety of the community itself. Most scientists have never heard of the Panton Principles, or the Open Knowledge Definition, and many adopt a rule of thumb that if the data are on the web and without cost, then that’s open enough for them. This is informal openness, and it is precisely this informal laissez faire attitude that renders the ecosystem fragile.

Informal openness is in fact quite threatening to the creation of downstream data works and products, because an upstream data owner retains the right to sue users for infringement. Formal open licensing removes that power, and provides clear freedom to operate for data users.

For example, the majority of chemical structure SDF files downloadable from chemical vendor websites have no defined licenses at all. Despite the assumptions that

PubChem data are “Open”, since the data are downloadable, they are not provided with any specific licenses per se but rather depositors assign rights simply by depositing data, thereby indicating acceptance of the depositors agreement. It is unlikely that the majority of scientists who download the data (possibly also for use in computational QSAR models) are aware of any license limitations constraining the data usage and have not concerned themselves with whether it is appropriate to monetize the data or repackage and redistribute under new licenses. It is just as unlikely that all depositors have fully understood that their data can be downloaded, redistributed and, ultimately, licensed, commoditized and monetized.

The ChEMBL database hosted by the European Bioinformatics Institute was recently released under a Creative Commons data license [20] and it is hoped that more databases will be released with such transparency in the future. That said, even such well-defined and community accepted licenses can be abused. The continuation of an original license through other aggregators is also difficult to police and the deposition of ChEMBL data to PubChem is made under the PubChem data transfer agreement [21] and will likely confuse the majority of the community to believing, once again, that all data are public domain, therefore requiring no attribution.

In the absence of specific data licensing terms, many data sources must assume to be licensed under the general terms of use of the websites from which they are available. These terms are often drafted without thought of the complexities of data, or to create legal certainty on data linked from websites, but instead carry boilerplate language to

prevent copyright infringement on text and images, or to limit liability for content contributed by third parties. This approach contributes significantly to the informality and legal uncertainty of data sourced from the web.

One attempt to address the lack of clarity is the “Is It Open” data project [22], in which direct questions are posed to data owners, and responses are posted on the web. But with only fifteen enquiries posted in the last twelve months, this project does not appear to be scaling with our ability to generate data.

If we do receive the guidance that a data set is “open,” we have more fundamental questions of data usage and citation to figure out. What does attribution mean in a data centric, cloud based world? What does share-alike mean in the context of data added to a database? Is it new data that we must share alike? Is it computational models that leverage the data? Is it metadata added to existing data? Is it the results of queries to a database API? What about annotations to data that live in a different place on the web and simply point to the data?

At present we really do not fully comprehend what it means yet to endlessly recombine data even though, in many ways, this activity is already underway in a number of the life sciences databases mentioned in this article. For example, both PubChem and ChemSpider have assimilated many tens of data sources from various depositors, each with their own licenses or, in most cases, undefined licenses. We do not know what the final result will be if indeed there is a single endpoint for the data. We do not endlessly

recombine creative works, although mash-ups of music and art appear to be a relatively recent phenomenon, driven by available technologies.

Once we see large scale scientific data mashups through integration of disparate databases and the result of these is seen to result in profit for someone, then attention may shift to the licenses behind the data. Although some would argue that data is perhaps not even property. The Open PHACTS project [23,24], for one, is combining data in a manner that will produce a single combined data set, for redistribution and download across multiple organizations and, necessarily, appropriate caution is being taken with the selection of data for inclusion. The project cannot afford to fall foul of ill-defined data licensing schemes.

The definitions and tools that we presently have to describe data are not well fitted to our needs. The Open Knowledge Foundation definitions are defined in regards to intellectual property protection. The Science Commons [25,26] protocols are stringent and challenging to embrace. The Creative Commons [27] licenses are focused on copyright protection, though the next global revisions of the licenses promise to address database rights as well. Perhaps most important, all of these definitions focus on the generation of reproductions and this is less and less relevant in a cloud-based world, where the copying of a dataset may be far less important than the querying of a dataset. So what happens when copying is no longer an act that triggers legal obligations of openness?

However complex the problem may be, and however ill-fitted the solution set, giving up is not an option. We must therefore begin to frame a set of practices that can stabilize the fragile ecosystem of data in drug discovery, one that is both standardized enough to allow for recombinant reuse and flexible enough to survive implementation in the real world.

References

1. Weininger D (1988) SMILES 1. Introduction and encoding rules. J Chem Inf Comput Sci 28: 31.
2. FigShare, http://figshare.com/
3. Flickr, http://www.flickr.com/
4. Chichester C, Mons B (2011) Collaboration and the semantic web. In: Ekins S, Hupcey MAZ, Williams AJ, editors. Collaborative computational technologies for biomedical research. Hoboken: John Wiley and Sons. pp. 453-466.
5. Ekins S, Clark AM, Williams AJ (2012) Open Drug Discovery Teams: A Chemistry Mobile App for Collaboration. Molecular Informatics, In Press, DOI: 10.1002/minf.201200034
6. Gamo F-J, Sanz LM, Vidal J, de Cozar C, Alvarez E, et al. (2010) Thousands of chemical starting points for antimalarial lead identification. Nature 465: 305-310.
7. GSK case study on Creative Commons, http://wiki.creativecommons.org/Case_Studies/GlaxoSmithKline
8. TB Commons, http://www.tbcommons.org/
9. Szalma S, Koka V, Khasanova T, Perakslis ED Effective knowledge management in translational medicine. J Transl Med 8: 68.
10. Szalma S, editor (2011) Enabling precompetitive translational research: a case study. Hoboken: John Wiley and Sons. 241-260 p.

11. Waller CL, Duravasula RV, Lynch N (2011) The need for collaborative technologies in drug discovery. In: Ekins S, Hupcey MAZ, Williams AJ, editors. Collaborative computational technologies for biomedical research. Hoboken, NJ: Wiley and Sons.

12. Barnes MR, Harland L, Foord SM, Hall MD, Dix I, et al. (2009) Lowering industry firewalls: pre-competitive informatics initiatives in drug discovery. Nat Rev Drug Discov 8: 701-708.

13. Creative Commons ShareAlike, http://creativecommons.org/licenses/by-sa/2.0/

14. US government data, http://data.gov

15. The Panton Principles, http://pantonprinciples.org/

16. Open definition, http://opendefinition.org/

17. Creative Commons Non-Commercial or No-Derivatives license, http://creativecommons.org/licenses/by-nc-nd/3.0/

18. Wilbanks J (2011) Openness as infrastructure. J Cheminform 3: 36.

19. Bunin BA, Ekins S (2011) Alternative business models for drug discovery. Drug Disc Today 16: 643-645.

20. ChEMBL Creative Commons Case Study, http://wiki.creativecommons.org/Case_Studies/ChEMBL

21. PubChem data transfer agreement, http://pubchem.ncbi.nlm.nih.gov/deposit/docs/PubChem_Data_Agreement.pdf

22. Is it open data? , http://www.isitopendata.org/

23. OpenPHACTS, http://www.openphacts.org/

24. Azzaoui K, Jacoby E, Senger S, Rodríguez EC, Loza M, et al. (2012) Analysis of the scientific competency questions followed by the IMI OpenPHACTS consortium for the development of the semantic web-based molecular information system OPS. Drug Disc Today, In Press.

25. Science Commons Empirical Data About Materials Transfer Problems, http://sciencecommons.org/projects/licensing/empirical-data-about-materials-transfer/

26. Nguyen T (2007) Science Commons: Material Transfer Agreement Project. Innovations: Technology, Governance, Globalization 2: 137-143.

27. Creative Commons Materials Transfer Project, http://sciencecommons.org/projects/licensing
